# Modelling the Effect of a Novel Autodissemination Trap on the Spread of Dengue in Shah Alam and Malaysia

**DOI:** 10.1155/2019/1923479

**Published:** 2019-08-04

**Authors:** Y. Liang, M. N. Ahmad Mohiddin, R. Bahauddin, F. O. Hidayatul, W. A. Nazni, H. L. Lee, D. Greenhalgh

**Affiliations:** ^1^Department of Mathematics and Statistics, University of Strathclyde, Glasgow G1 1XH, UK; ^2^School of Diagnostic and Applied Health Sciences, Faculty of Health Sciences, Universiti Kebangsaan Malaysia, Jalan Raja Muda A. Aziz, 50300 Kuala Lumpur, Malaysia; ^3^Medical Entomology Unit, Institute for Medical Research, Jalan Pahang, 50588 Kuala Lumpur, Malaysia; ^4^Vector Borne Disease Control Branch, Disease Control Division, Ministry of Health, Putrajaya, Malaysia

## Abstract

In this paper, we will start off by introducing the classical Ross–Macdonald model for vector-borne diseases which we use to describe the transmission of dengue between humans and *Aedes* mosquitoes in Shah Alam, which is a city and the state capital of Selangor, Malaysia. We will focus on analysing the effect of using the Mosquito Home System (MHS), which is an example of an autodissemination trap, in reducing the number of dengue cases by changing the Ross–Macdonald model. By using the national dengue data from Malaysia, we are able to estimate *λ*, which represents the initial growth rate of the dengue epidemic, and this allows us to estimate the number of mosquitoes in Malaysia. A mathematical expression is also constructed which allows us to estimate the potential number of breeding sites of *Aedes* mosquitoes. By using the data available from the MHS trial carried out in Section 15 of Shah Alam, we included the potential effect of the MHS into the dengue model and thus modelled the impact MHS has on the spread of dengue within the trial area. We then extended our results to analyse the effect of the MHSs on reducing the number of dengue cases in the whole of Malaysia. A new model was constructed with a basic reproduction number, *R*_0,Mala_^MHS^, which allows us to identify the required MHSs coverage needed to achieve extinction in Malaysia. Numerical simulations and tables of results were also produced to illustrate our results.

## 1. Introduction

Epidemics of infectious diseases have been a constant threat towards our society. In the past, Europe suffered from 25 million deaths out of a population of 100 million due to the Black Death [1]; Russia suffered from about 25 million cases of typhus with a death rate of about 10 percent, whilst smallpox wiped out half of the population of the Aztecs of three and a half million in 1520 [2]. Although in the 21st century, many diseases such as smallpox no longer pose a threat towards mankind, but there is still a high proportion of the population that is under threat of diseases such as malaria and dengue. According to the World Health Organization, every year there are around 50–100 million dengue infections where at least 100 countries have a dengue epidemic [3]. Dengue is a vector-borne disease which is transmitted by the *Aedes* mosquitoes which are also responsible for the transmission of yellow fever and the Zika virus [3, 4].

Malaysia, a country in the Southeast of Asia, has consistently been reported to have a high number of dengue cases due to its tropical climate. Between 2014 and 2016, Malaysia had around 330,891 reported dengue cases with around 788 dengue-related deaths with a high incidence rate of 396.4 per 100,000 population in 2015 causing it to suffer from serious economic and health burdens. In the study of Packierisamy et al. [5], it is estimated that, in 2010, it had cost Malaysia around USD $73.45 million in dengue-related vector control which was around USD $2.63 per capita population. The standard and traditional way of battling against dengue is by using space spraying (chemical fogging); however, the effect tends to reduce over time [6]. In addition, over time, it is possible for the *Aedes* mosquitoes to survive and develop resistance to the chemical that is used in space spraying [7] which reduces the effectiveness of spraying in controlling the spread of dengue. An alternative way by which we can combat dengue is by using the autodissemination trap [6], which is a more proactive method as the trap contains a special solution which will lure the female *Aedes* mosquitoes to lay eggs inside the trap. Most importantly, the eggs that are laid will get killed off by the solution inside thus preventing them from hatching into adult *Aedes* mosquitoes to transmit the disease. As a result, the autodissemination trap will essentially reduce the *Aedes* population size.

In this paper, we will modify the classic Ross–Macdonald dengue model [8] to examine the effect of such an autodissemination trap called the Mosquito Home System (MHS) in controlling the spread of dengue. The MHS data used in this paper are collected from the site of the trial that took place in an environment consisting of shop houses in Section 15 of Shah Alam, the state capital of the highly dengue infected area, Selangor, Malaysia.

This paper is arranged as follows: In Section 2, we will introduce the classical Ross–Macdonald model and the basic reproduction number. We then modify the Ross–Macdonald model to twelve differential equation models which describe the spread of dengue between humans and *Aedes* mosquitoes both in Malaysia and in the trial site in Section 15 of Shah Alam, Selangor, Malaysia. We will also construct a list of different biting proportions corresponding to different times spent outside the trial site. In Section 3, we will perform thorough analysis on the effect of having different levels of MHSs on the number of dengue cases in the trial site in Shah Alam. In Section 4, we extend our results from the trial site in Shah Alam to the whole of Malaysia. A new improved model is constructed with a new basic reproduction number. The extinction condition is also derived. Lastly, in Section 5, we summarise our results. Numerical simulations produced using Euler's method and tables of results are shown throughout this paper.

## 2. The Modified Ross–Macdonald Dengue Model with the Effect of Autodissemination Trap

Let us start by introducing the delayed Ross–Macdonald SIR model for dengue used in [8, 9] which our modified dengue model will be based on

(1) $$\begin{array}{c} \frac{dS_{\text{H}}\left(t\right)}{dt}=-abI_{\text{v}}\left(t\right)\frac{S_{\text{H}}\left(t\right)}{N_{\text{H}}}-\mu_{\text{H}}S_{\text{H}}\left(t\right)+\mu_{\text{H}}N_{\text{H}},\\ \frac{dI_{\text{H}}\left(t\right)}{dt}=abI_{\text{v}}\left(t\right)\frac{S_{\text{H}}\left(t\right)}{N_{\text{H}}}-\left(\mu_{\text{H}}+\gamma \right)I_{\text{H}}\left(t\right),\\ \frac{dR_{\text{H}}\left(t\right)}{dt}=\gamma I_{\text{H}}\left(t\right)-\mu_{\text{H}}R_{\text{H}}\left(t\right),\\ \frac{dS_{\text{v}}\left(t\right)}{dt}=-acS_{\text{v}}\left(t\right)\frac{I_{\text{H}}\left(t\right)}{N_{\text{H}}}-\mu_{\text{v}}S_{\text{v}}\left(t\right)+\mu_{\text{v}}N_{\text{v}},\\ \frac{dL_{\text{v}}\left(t\right)}{dt}=acS_{\text{v}}\left(t\right)\frac{I_{\text{H}}\left(t\right)}{N_{\text{H}}}-\mu_{\text{v}}L_{\text{v}}\left(t\right)-acS_{\text{v}}\left(t-\tau \right)\frac{I_{\text{H}}\left(t-\tau \right)}{N_{\text{H}}}e^{-\mu_{\text{v}}\tau },\\ \frac{dI_{\text{v}}\left(t\right)}{dt}=acS_{\text{v}}\left(t-\tau \right)\frac{I_{\text{H}}\left(t-\tau \right)}{N_{\text{H}}}e^{-\mu_{\text{v}}\tau }-\mu_{\text{v}}I_{\text{v}}\left(t\right), \end{array}$$

with initial conditions *S*_H_(0), *I*_H_(0), and *R*_H_(0). *S*_H_(*t*), *I*_H_(*t*), and *R*_H_(*t*) represent, respectively, the susceptible, infected, and recovered humans, while *S*_v_(0), *L*_v_(0), and *I*_v_(0) denote the initial conditions for *S*_v_(*t*), *L*_v_(*t*), and *I*_v_(*t*) which represent, respectively, the susceptible, latent, and infected mosquitoes. Note that *N*_H_=*S*_H_+*I*_H_+*R*_H_ denotes the total population size for humans and *N*_v_=*S*_v_+*L*_v_+*I*_v_ represents the total population for *Aedes* mosquitoes, both constant. The biological meanings of the parameter values used in equation (1) are given in Table 1.

Note that the delayed Ross–Macdonald SIR model only includes the extrinsic incubation period in mosquitoes, whereas actually sometimes an intrinsic incubation period in humans is also included in the model. However, it is important to note that much work has already been performed on dengue models with just extrinsic incubation period only [9–15]. Therefore, we hope that the results mentioned in this paper will be able to contribute to this research area.

There are two different conventions for defining the basic reproduction number *R*_0_ in host-vector models. In this paper, we are using the convention that a generation of disease transmission is human to human disease transmission. Thus, *R*_0_ is defined as the expected number of secondary cases in humans caused by a single newly infected human entering a disease-free population at equilibrium [8, 9, 13–16]. A secondary case is defined as a person directly infected by a mosquito which was directly infected by the original infected individual. However, the next-generation matrix approach [16–21] effectively regards a generation of disease transmission to be either human to mosquito transmission or mosquito to human transmission. With this approach, the new basic reproduction number, ${\tilde{R}}_{0}$ say, is the square root of our *R*_0_. This ${\tilde{R}}_{0}$ has the same threshold value as the one which we have derived (i.e., ${\tilde{R}}_{0}>1$ , if and only if *R*_0_ > 1, and ${\tilde{R}}_{0}<1$ , if and only if *R*_0_ < 1).

For example, if we take the delayed Ross–Macdonald SIR model for dengue given in equation (1), if a newly infectious human enters a disease-free population at equilibrium, he or she will remain infectious for time

(2) $$\begin{array}{c} \frac{1}{\mu_{\text{H}}+\gamma }, \end{array}$$

and during this time infect

(3) $$\begin{array}{c} \frac{N_{\text{v}}}{N_{\text{H}}}\frac{ac}{\mu_{\text{H}}+\gamma }=\frac{mac}{\mu_{\text{H}}+\gamma } \end{array}$$

mosquitoes. Here, *m*=*N*_v_/*N*_H_ represents the number of *Aedes* mosquitoes per human. A fraction *e* ^−*μ*_*v*_ *τ*^ of these survives the latent period to become infectious; thus, there are

(4) $$\begin{array}{c} \frac{mac}{\mu_{\text{H}}+\gamma }e^{-\mu_{\text{v}}\tau } \end{array}$$

infectious mosquitoes.

Using a similar argument, each newly infectious mosquito directly infects

(5) $$\begin{array}{c} \frac{ab}{\mu_{\text{v}}} \end{array}$$

humans. Thus,

(6) $$\begin{array}{c} R_{0}=\frac{mac}{\mu_{\text{H}}+\gamma }e^{-\mu_{\text{v}}\tau }\frac{ab}{\mu_{\text{v}}}. \end{array}$$

Therefore,

(7) $$\begin{array}{c} R_{0}=\frac{ma^{2}bce^{-\mu_{\text{v}}\tau }}{\mu_{v}\left(\mu_{\text{H}}+\gamma \right)}. \end{array}$$

Note also that our differential equation would reduce to a standard SIR-SI case if *τ*=0 in equation (7).

On the contrary, using the next-generation matrix approach, there are three types of infectious individuals: namely, infectious humans (*i* = 1), latent mosquitoes (*i* = 2), and infectious mosquitoes (*i* = 3). The next-generation matrix is defined as the matrix **M**={*M*_*ij*_ : *i*=1,2,3, *j*=1,2,3}, where *M*_*ij*_ is defined to be the number of secondary cases in infectious state *i* caused by a single newly infected individual in infectious state *j* entering a disease-free population at equilibrium. Hence,


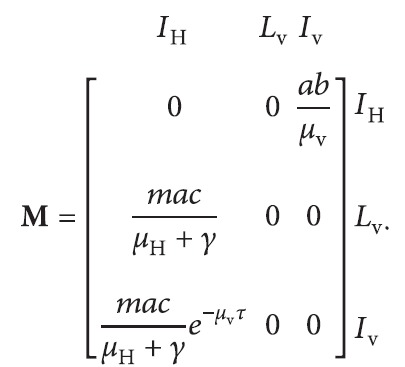

(8)



It is straightforward to show that the eigenvalues of this matrix are *ω*=0 and $\omega =\pm \sqrt{R_{0}}$ , so the basic reproduction number calculated using the next-generation matrix method is the largest absolute value of these, which is ${\tilde{R}}_{0}=\sqrt{R_{0}}$ .

The estimation of *λ*, the initial per capita growth rate of the dengue epidemic, is outlined in detail in [12], but we summarise it here for convenience. It is easy to obtain values for some of the quantities in equation (1). For example, 1/*γ* is the average human infectious period, 1/*μ*_v_ represents the average mosquito lifetime, and 1/*μ*_H_ is the average human lifetime. However, the total number of mosquitoes, *N*_v_, is more difficult to estimate. At the start, the number of dengue cases is expected to grow exponentially such as *e* ^*λt*^. We therefore estimate *λ* by fitting a function *Ae* ^*λt*^, where *A* is a constant, to the initial numbers of infected humans. At the start, where this fitting was done, the initial number of infected individuals ranged from 390 to 2,468. In other words, the infected fraction of the total Malaysian population of 32,000,000 [22] ranged from 1.22 × 10^−5^ to 7.71 × 10^−5^. In this case, the number of infected individuals, latent mosquitoes, and infected mosquitoes behave in a similar manner where *I*_H_(*t*), *L*_v_(*t*), and *I*_v_(*t*) grow exponentially at the same rate over a short period of time, namely, *I*_H_(*t*) ≈ *I*_H_(0)*e* ^*λt*^, *L*_v_(*t*) ≈ *L*_v_(0)*e* ^*λt*^, and *I*_v_(*t*) ≈ *I*_v_(0)*e* ^*λt*^ [23], where *I*_H_(0), *L*_v_(0), and *I*_v_(0) are the initial conditions for the number of infected individuals, latent mosquitoes, and infected mosquitoes, respectively.

As described in [12], by plotting an exponential curve fitted to the real dengue cases in humans in Malaysia (available in [24]), we obtain the fitted initial per capita growth rate *λ* of the dengue cases. We obtain *λ* = 0.00053/day.

From this *λ* estimate, we can find the basic reproduction number *R*_0_, and from *R*_0_, we can estimate *N*_v_ (recall that *R*_0_ represents the expected number of secondary cases in humans that will arise from a single infected person entering a disease-free population at equilibrium. Alternatively, *R*_0_ can be thought of as the expected number of secondary cases in mosquitoes that will arise from a single infected mosquito entering a disease-free population at equilibrium).

The essential method which we shall use follows the one outlined in Appendix of [14]. In [14], *λ* is estimated from the initial phase of the epidemic, and then, *R*_0_ is estimated from the parameters *λ*,  *μ*_H_,  *μ*_v_,  *τ*, and *γ*, all of which we know either from data or the literature. Equation (11) gives our *R*_0_ estimate for the model (1) obtained using this technique. Equation (7) is found directly using the model (1) and gives *R*_0_ in terms of the model parameters. We chose to estimate *R*_0_ using equation (11) rather than using equation (7) because it is difficult to estimate *N*_v_, the total number of mosquitoes in Malaysia, and hence difficult to estimate *m*=*N*_v_/*N*_H_, the number of mosquitoes per human.

We obtain equation (11) by following the argument in Appendix of [14]. However, we observe that, in the second equation, in both of equations (9) and (10) in [14], the argument of *i*_*H*_ should be *t* − *τ* as opposed to *t*. Noting this, the argument for that model does not give

(9) $$\begin{array}{c} R_{0}=\left(1+\frac{\lambda^{2}+\lambda \left(\mu +\gamma \right)}{\mu \gamma }\right), \end{array}$$

as stated in [14], but instead

(10) $$\begin{array}{c} R_{0}=\left(1+\frac{\lambda^{2}+\lambda \left(\mu +\gamma \right)}{\mu \gamma }\right)e^{\lambda \tau } \end{array}$$

as given in [12]. *μ* in equations (9) and (10) is the per capita mosquito death rate, which in our model is denoted *μ*_v_.

We apply the same method to our model. Our model includes extra parameters, namely, the disease transmission probability to an uninfected mosquito from an infectious human at each bite, which is *c*, and the death rate per human, which is *μ*_H_. We find that, for our model,

(11) $$\begin{array}{c} R_{0}=\frac{\left(\lambda +\mu_{\text{v}}\right)\left(\lambda +\mu_{\text{H}}+\gamma \right)e^{\lambda \tau }}{\left(\mu_{\text{H}}+\gamma \right)\mu_{\text{v}}}. \end{array}$$

In this paper, our aim is to analyse the effect of using the MHSs on reducing the number of dengue cases in the trial site in Section 15 of Shah Alam, Selangor, Malaysia, and thus later extend the analysis to the whole of Malaysia. Note that the trial site consists of fifteen blocks of shop houses. Therefore, we will modify the six differential equations delayed dengue model to a twelve differential equations dengue model where six of them describe the disease dynamics in Malaysia, while the other six represent the dynamics of dengue within the trial site in Shah Alam with the effect of MHSs. The modified dengue model is given as follows:

(12) $$\begin{array}{c} \frac{dS_{{\text{H}}_{1}}\left(t\right)}{dt}=-abI_{{\text{v}}_{1}}\left(t\right)\frac{S_{{\text{H}}_{1}}\left(t\right)}{N_{{\text{H}}_{1}}}-\mu_{\text{H}}S_{{\text{H}}_{1}}\left(t\right)+\mu_{\text{H}}N_{{\text{H}}_{1}},\\ \frac{dI_{{\text{H}}_{1}}\left(t\right)}{dt}=abI_{{\text{v}}_{1}}\left(t\right)\frac{S_{{\text{H}}_{1}}\left(t\right)}{N_{{\text{H}}_{1}}}-\left(\mu_{\text{H}}+\gamma \right)I_{{\text{H}}_{1}}\left(t\right),\\ \frac{dR_{{\text{H}}_{1}}\left(t\right)}{dt}=\gamma I_{{\text{H}}_{1}}\left(t\right)-\mu_{\text{H}}R_{{\text{H}}_{1}}\left(t\right),\\ \frac{dS_{{\text{v}}_{1}}\left(t\right)}{dt}=-acS_{{\text{v}}_{1}}\left(t\right)\frac{I_{{\text{H}}_{1}}\left(t\right)}{N_{{\text{H}}_{1}}}-\mu_{\text{v}}S_{{\text{v}}_{1}}\left(t\right)+\mu_{\text{v}}N_{{\text{v}}_{1}},\\ \frac{dL_{{\text{v}}_{1}}\left(t\right)}{dt}=acS_{{\text{v}}_{1}}\left(t\right)\frac{I_{{\text{H}}_{1}}\left(t\right)}{N_{{\text{H}}_{1}}}-\mu_{\text{v}}L_{{\text{v}}_{1}}\left(t\right)-acS_{{\text{v}}_{1}}\left(t-\tau \right)\frac{I_{{\text{H}}_{1}}\left(t-\tau \right)}{N_{{\text{H}}_{1}}}e^{-\mu_{\text{v}}\tau },\\ \frac{dI_{{\text{v}}_{1}}\left(t\right)}{dt}=acS_{{\text{v}}_{1}}\left(t-\tau \right)\frac{I_{{\text{H}}_{1}}\left(t-\tau \right)}{N_{{\text{H}}_{1}}}e^{-\mu_{\text{v}}\tau }-\mu_{\text{v}}I_{{\text{v}}_{1}}\left(t\right), \end{array}$$

(13) $$\begin{array}{c} \frac{dS_{{\text{H}}_{2}}\left(t\right)}{dt}=-ab\left(P\frac{I_{{\text{v}}_{1}}\left(t\right)}{N_{{\text{H}}_{1}}}+\left(1-P\right)\frac{I_{{\text{v}}_{2}}\left(t\right)}{N_{{\text{H}}_{2}}}\right)S_{{\text{H}}_{2}}\left(t\right)-\mu_{\text{H}}S_{{\text{H}}_{2}}\left(t\right)+\mu_{\text{H}}N_{{\text{H}}_{2}},\\ \frac{dI_{{\text{H}}_{2}}\left(t\right)}{dt}=ab\left(P\frac{I_{{\text{v}}_{1}}\left(t\right)}{N_{{\text{H}}_{1}}}+\left(1-P\right)\frac{I_{{\text{v}}_{2}}\left(t\right)}{N_{{\text{H}}_{2}}}\right)S_{{\text{H}}_{2}}\left(t\right)-\left(\mu_{\text{H}}+\gamma \right)I_{{\text{H}}_{2}}\left(t\right),\\ \frac{dR_{{\text{H}}_{2}}\left(t\right)}{dt}=\gamma I_{{\text{H}}_{2}}\left(t\right)-\mu_{\text{H}}R_{{\text{H}}_{2}}\left(t\right),\\ \frac{dS_{{\text{v}}_{2}}\left(t\right)}{dt}=-acS_{{\text{v}}_{2}}\left(t\right)\frac{I_{{\text{H}}_{2}}\left(t\right)}{N_{{\text{H}}_{2}}}-\mu_{\text{v}}S_{{\text{v}}_{2}}\left(t\right)+\mu_{\text{v}}N_{{\text{v}}_{2}}\left(1-P^{*}\right),\\ \frac{dL_{{\text{v}}_{2}}\left(t\right)}{dt}=acS_{{\text{v}}_{2}}\left(t\right)\frac{I_{{\text{H}}_{2}}\left(t\right)}{N_{{\text{H}}_{2}}}-\mu_{\text{v}}L_{{\text{v}}_{2}}\left(t\right)-acS_{{\text{v}}_{2}}\left(t-\tau \right)\frac{I_{{\text{H}}_{2}}\left(t-\tau \right)}{N_{H_{2}}}e^{-\mu_{\text{v}}\tau },\\ \frac{dI_{{\text{v}}_{2}}\left(t\right)}{dt}=acS_{{\text{v}}_{2}}\left(t-\tau \right)\frac{I_{{\text{H}}_{2}}\left(t-\tau \right)}{N_{{\text{H}}_{2}}}e^{-\mu_{\text{v}}\tau }-\mu_{\text{v}}I_{{\text{v}}_{2}}\left(t\right), \end{array}$$

where all the parameter values are defined as before in Table 1. Note that subscript (·)_1_ refers to Malaysia, while subscript (·)_2_ refers to the trial site in Section 15 of Shah Alam, Selangor, Malaysia. *P* ^*∗*^ represents the proportion reduction in the *Aedes* population as a result of using the MHSs in the trial site in Shah Alam which is the key parameter value in deciding the effectiveness of the MHSs. *P* represents the proportion of all mosquito bites experienced by an individual whilst the person is outside the trial site. So 1 − *P* represents the proportions of all bites experienced by a person which are experienced while the person is inside the trial site.

We can derive the basic reproduction number for the modified model given by using equations (12) and (13) either by using the next-generation matrix method with infectious individuals being either infectious humans or latent or infectious mosquitoes, or alternatively by adapting this method so that the infectious individuals are only infectious humans. To start with the first of these approaches, there are six types of infectious individuals: namely, infectious humans outside the trial site (*i* = 1), latent mosquitoes outside the trial site (*i* = 2), infectious mosquitoes outside the trial site (*i* = 3), infectious humans inside the trial site (*i* = 4), latent mosquitoes inside the trial site (*i* = 5), and infectious mosquitoes inside the trial site (*i* = 6). The next-generation matrix is


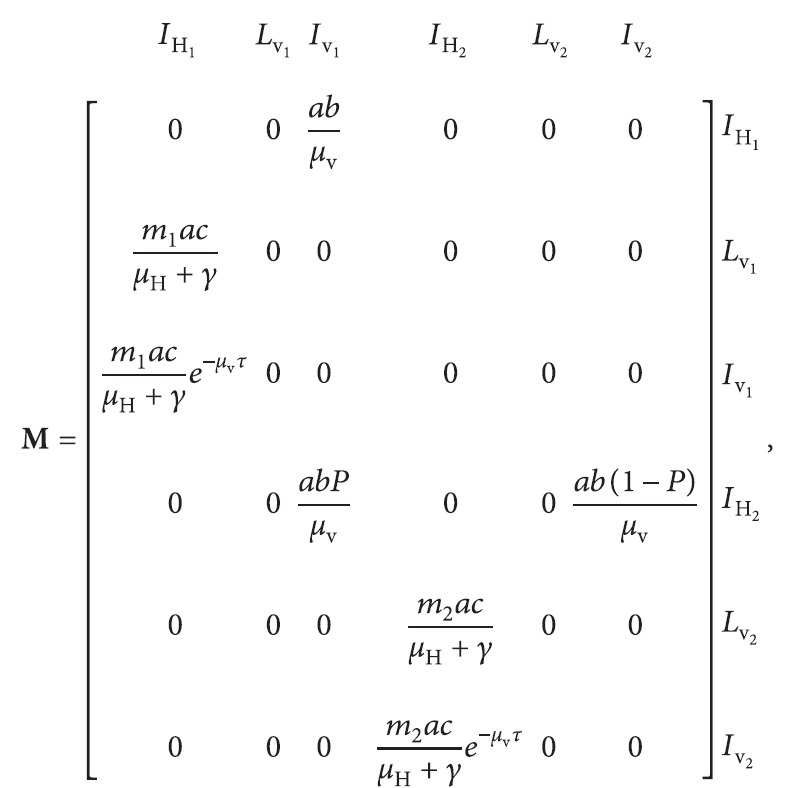

(14)
where *m*_1_=*N*_v_1__/*N*_H_1__ is the ratio of mosquitoes to humans outside the trial site and *m*_2_=*N*_v_2__(1 − *P* ^*∗*^)/*N*_H_2__ is the ratio of mosquitoes to humans inside the trial site in the presence of the MHSs. It is straightforward to show that the eigenvalues of this matrix are

(15) $$\begin{array}{c} \omega =0,\;\left(\text{twice}\right),\\ \omega =\pm \sqrt{\frac{m_{1}a^{2}bce^{-\mu_{\text{v}}\tau }}{\mu_{\text{v}}\left(\mu_{\text{H}}+\gamma \right)}},\\ \omega =\pm \sqrt{\frac{m_{2}a^{2}bce^{-\mu_{\text{v}}\tau }\left(1-P\right)}{\mu_{\text{v}}\left(\mu_{\text{H}}+\gamma \right)}}. \end{array}$$

We assume that, in the absence of the MHSs, the ratio of mosquitoes to humans is the same inside or outside the trial site, i.e.,

(16) $$\begin{array}{c} m_{1}=\frac{N_{{\text{v}}_{1}}}{N_{{\text{H}}_{1}}}=\frac{N_{{\text{v}}_{2}}}{N_{{\text{H}}_{2}}},\\ \text{then }m_{1}\geq m_{2}\left(1-P\right)=\frac{N_{{\text{v}}_{2}}}{N_{{\text{H}}_{2}}}\left(1-P^{*}\right)\left(1-P\right). \end{array}$$

So for the modified model given by using equations (12) and (13), the basic reproduction number ${\tilde{R}}_{0}^{1}$ calculated by the next-generation matrix method is ${\tilde{R}}_{0}^{1}={\tilde{R}}_{0}$ , the same as for the delayed Ross–Macdonald model (1). In the rest of the paper, we prefer to use the definition of the basic reproduction number given by counting human to human disease transmission as one generation as it corresponds to our previous work, the work of Massad et al. and others [8, 9, 13–16]. With this definition, the basic reproduction number for the modified model is *R*_0_, again the same as that for the Ross–Macdonald model. It is possible to show this directly by constructing an argument similar to the next-generation matrix argument given above but with infectious individuals corresponding only to the two types of infectious humans outside and inside the trial site. In this case, the corresponding next-generation matrix has eigenvalues *R*_0_ and *R*_0_(1 − *P* ^*∗*^)(1 − *P*). Hence, the largest absolute eigenvalue is *R*_0_, which is the basic reproduction number for the modified models (12) and (13).

Note that *Aedes* mosquitoes are more likely to bite at dawn and dusk. In Table 2, we have five different daily time slots in which a person decides to leave the trial site in Shah Alam and be in the rest of Malaysia. The proportion of the total number of bites experienced by this person which are experienced outside the trial site is *P*. Thus, the corresponding proportion of the total number of bites experienced inside the trial site is 1 − *P*. These are got by integrating data for mosquito biting rates at different times of day over the relevant time period [25]. Before we begin our analysis, it is important for us to know all the required parameter values.

The required parameter values for Malaysia and the trial site in Section 15 of Shah Alam, Selangor, Malaysia, are given in Table 3. Note that *m* represents the number of *Aedes* mosquitoes per person. From [29], *m* ranges from 0.34 to 22.7 depending on the location; thus in this paper, we have chosen *m* to be around 1.867 which is close to the estimation for Thailand [29].

Note also that the total human population in the trial site area, *N*_H_2__, is estimated using the information in [30]. The MHS trial site in which we are interested is located within Section 15 of Shah Alam, where according to [30], the area is highly densely populated and undergoing rapid development with an estimate of around 2,000 residents per km^2^. Our trial site is approximately 4.320 km^2^; therefore, *N*_H_2__ is around 8,640.

### 2.1. Estimating the Number of *Aedes* Breeding Sites

Before we start our analysis, it would be useful to know the number of potential breeding sites for *Aedes* mosquitoes as this could help us in determining the appropriate number of MHSs that would be needed in order to achieve a substantial effect in reducing the number of dengue cases.

In the development of the MHSs, a trial was carried out in Singapore Botanic Gardens (Jacob Ballas Children's Park) [12]. The amount of eggs obtained was counted both in special ovitraps, called Gravitraps, and the MHSs. Here, in the Gravitraps, there were twelve times fewer eggs than in the MHSs. The MHSs and the Gravitraps were the only mosquito breeding sites where the number of eggs was known. Hence, we suppose that mosquitoes lay a factor of twelve times as many eggs in each MHS as in each other possible breeding site. The MHSs contain a particular solution in which *Aedes* mosquitoes prefer to lay eggs. So the MHSs are expected to collect more eggs than the other possible breeding sites.

Let us assume that there are *x* MHSs being deployed, *o* ovitraps, and *y* hidden breeding sites. Hence, the probability that an egg is laid in an ovitrap is

(17) $$\begin{array}{c} \frac{o}{12x+o+y}. \end{array}$$

As there are *k* eggs altogether, the total expected number of eggs laid in all of the ovitraps taken together is

(18) $$\begin{array}{c} \frac{ko}{12x+o+y}. \end{array}$$

So this is the expected number of eggs collected by the ovitraps within the trial site. Note that the formulation for equation (18) is obtained by following a similar idea illustrated in [12]. Thus, the number of eggs per ovitrap is

(19) $$\begin{array}{c} \frac{1}{o}\frac{ko}{12x+o+y}=\frac{k}{12x+o+y}. \end{array}$$

This will be used in equations (20) and (21) to estimate the number of hidden breeding sites. From the data collected from the trial site in Section 15 in Shah Alam, we have that the mean number of eggs collected per ovitrap decreased by a factor of 0.7723 from 29.74 to 22.97 eggs per trap after they increased the number of MHSs from 340 to 625. Therefore, the number of hidden breeding sites can be estimated by comparing the percentage reduction in the mean number of eggs per ovitrap before and after they increased the number of MHSs. As a result, the number of hidden breeding sites can be estimated as follows:

(20) $$\begin{array}{c} \frac{0.7723k}{12\times 340+126+y}=\frac{k}{12\times 625+193+y}, \end{array}$$

where the number of ovitraps have also increased accordingly to cover more blocks of shop houses (from 126 to 193). Hence,

(21) $$\begin{array}{c} \frac{0.7723}{12\times 340+126+y}=\frac{1}{12\times 625+193+y}. \end{array}$$

By rearranging equation (21) and solving for *y*, which represents the number of hidden sites, we have that *y*=7,621.

## 3. Effect of MHSs on Spread of Dengue in Trial Site in Section 15 of Shah Alam

In this section, we will focus on working with equation (13) which describes the dynamical behaviour of dengue within the 18 blocks of shop houses (the trial site) in Section 15 of Shah Alam, Selangor, Malaysia. As mentioned previously, the key parameter value that we wish to investigate is *P* ^*∗*^, which represents the proportion of reduction in the *Aedes* population as a result of the MHSs.

There are three different scenarios we will explore in this section, namely,

1. No MHSs within the 18 blocks of shop houses (the trial site)
2. 340 MHSs with 126 ovitraps within the 18 blocks of shop houses
3. 625 MHSs with 193 ovitraps within the 18 blocks of shop houses

By following a similar argument as in Section 3 of [12], *P* ^*∗*^ can be expressed by using the following equation:

(22) $$\begin{array}{c} P^{*}=\frac{12x}{12x+o+y}, \end{array}$$

where *x*, *o*, and *y* are defined as before. This can also be obtained from the argument in Section 2.1 by noting that *P* ^*∗*^ is the probability that an egg is laid in an MHS which is given by using equation (22). After substituting all the required parameter values into equation (22), we have that

1. *P* ^*∗*^=0, if there are no MHSs within the 18 blocks of shop houses
2. *P* ^*∗*^=0.3450, if we have 340 MHSs within the 18 blocks of shop houses
3. *P* ^*∗*^=0.4897, if we have 625 MHSs within the 18 blocks of shop houses

Note that the *P* ^*∗*^ values obtained from the Jacob Ballas Children's Park trial at Singapore Botanic Gardens (*P* ^*∗*^=0.5889) as well as from the trials carried out in 3 blocks of flats in Selangor [12] (*P* ^*∗*^=0.6871 and *P* ^*∗*^=0.7789) are higher than the ones we use in this paper.

Note also, unless stated otherwise, the unit of time is in weeks.

### 3.1. Numerical Simulation for Using MHSs in 18 Blocks of Shop Houses in Shah Alam

Let us define all the parameter values as before. The total population in Malaysia in 2017 was around 32 million [31]. The dengue incidence rate in Malaysia in 2017 was approximately 258.9 per 100,000 people [32]. Typically, dengue cases have a high ratio of asymptomatic to symptomatic cases of 4 to 1, respectively, and thus, we will increase the incidence rate by five times to reflect this situation. By using the dengue incidence rate in Malaysia in 2017, we can estimate the initial value for the number of infected individuals in Malaysia, namely, *I*_H_1__(0). In addition, from 1995 to 2016, the number of dengue cases in Malaysia was around 879,501 (without taking into consideration the asymptomatic cases). As a result, by using *S*(*t*)+*I*(*t*)+*R*(*t*)=*N* and the above information, the initial values, working to 3 d.p., of equations (12) and (13) are

(23) $$\begin{array}{c} S_{{\text{H}}_{1}}\left(0\right)=27,594.529,I_{{\text{H}}_{1}}\left(0\right)=7,966.154,R_{{\text{H}}_{1}}\left(0\right)=4,397.505,\\ S_{{\text{v}}_{1}}\left(0\right)=59,741.088,L_{{\text{v}}_{1}}\left(0\right)=6,797.226,I_{{\text{v}}_{1}}\left(0\right)=8,818.462,\\ S_{{\text{H}}_{2}}\left(0\right)=7,450.523,I_{{\text{H}}_{2}}\left(0\right)=2.151,R_{{\text{H}}_{2}}\left(0\right)=1,187.326,\\ S_{{\text{v}}_{2}}\left(0\right)=16,130.09,L_{{\text{v}}_{2}}\left(0\right)=1.835,I_{{\text{v}}_{2}}\left(0\right)=2.381, \end{array}$$

where we have set the initial values within the trial site to reflect the distribution of the initial values in Malaysia. Note that the initial conditions for the number of susceptible, latent, and infected mosquitoes in Malaysia are obtained using the initial value of *I*_*H*_1__(0) and *N*_*H*_1__ and substituting them into the equilibrium version of the differential equations for (*dS*_v_1__(*t*)/*dt*), (*dL*_*v*_1__(*t*)/*dt*), and (*dI*_*v*_1__(*t*)/*dt*).

#### 3.1.1. Example 1 (*P*=0.1280)

In this example, let us choose a small *P* value from Table 2, namely, 0.1280, to denote the proportion of bites a person will get if going outside the trial site between 9 a.m. and 4.45 p.m. as stated in Table 2.

**Figure 1:**
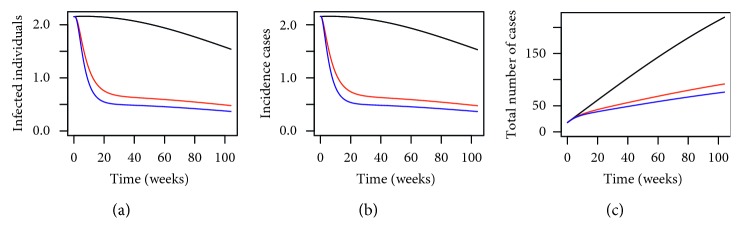
Numerical simulations for (a) infected individuals, (b) incidence cases (in weeks), and (c) cumulative total number of dengue cases within the trial site in Section 15 of Shah Alam, Selangor, Malaysia, for *P*=0.1280 where the black line, red line, and blue line represent *P* ^*∗*^=0, *P* ^*∗*^=0.3450, and *P* ^*∗*^=0.4897, respectively, over a period of 2 years.

By using the parameter values defined in Table 3 and the above initial conditions, we have the numerical simulation results given in Figure 1. From Figure 1, we can see that by deploying MHSs in the trial sites in Shah Alam, the number of infected individuals, incidence cases, and the total number of dengue cases have all reduced drastically. In addition, we can see that by having more MHSs (illustrated by using the blue lines), the effect of reducing the number of dengue cases is greater than having a lower number of MHSs (the red lines) which is to be expected. The numerical results given in Figure 1 are very promising as they demonstrate the effectiveness of using the MHSs in reducing the number of dengue cases in the trial area.

**Figure 2:**
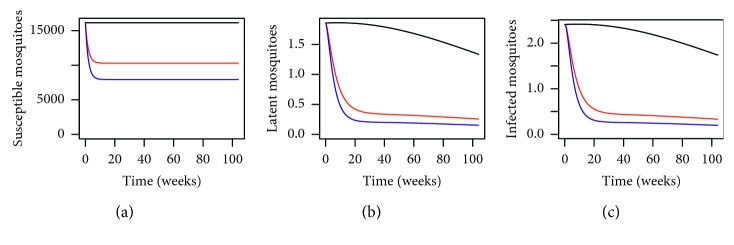
Dynamical behaviour for *Aedes* mosquitoes within the trial site in Section 15 of Shah Alam, Selangor, Malaysia, for *P*=0.1280 where the black line, red line, and blue line represent *P* ^*∗*^=0, *P* ^*∗*^=0.3450, and *P* ^*∗*^=0.4897, respectively, over a period of 2 years.

In order to analyse the impact of using the MHSs and understand better as to why we have a lower number of dengue cases when we use the MHSs, let us observe Figure 2. From Figure 2, we have the dynamical behaviour of *Aedes* mosquitoes for different levels of MHSs. By using the MHSs in the trial site in Section 15 of Shah Alam, we can see that the number of susceptible, infected, and latent mosquitoes have reduced drastically. This is again to be expected as the MHS is designed to lure the female *Aedes* mosquitoes to lay eggs there. Any eggs laid will be killed and will not hatch into adult *Aedes* to transmit dengue. By using the MHSs, the total *Aedes* population size will reduce thus reducing the number of dengue cases as illustrated here numerically.

In the next example, we will observe what will happen if we choose a higher *P* value, say *P*=0.6610. In other words, what will happen to the number of dengue cases if an average person stays outside the trial site for a longer period of time (and thus potentially has a greater chance of getting bitten more often). In this case, the longer period of time is between 6 a.m. and 7.45 p.m.

#### 3.1.2. Example 2 (*P*=0.6610)

**Figure 3:**
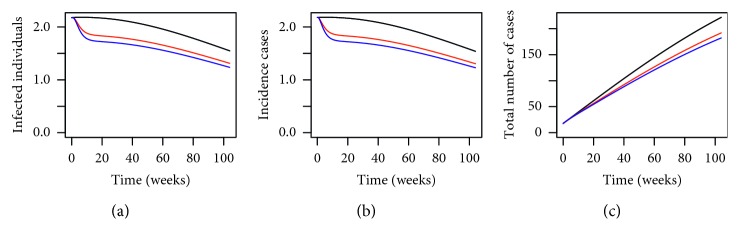
Numerical simulations for (a) infected individuals, (b) incidence cases (in weeks), and (c) cumulative total number of dengue cases within the trial site in Section 15 of Shah Alam, Selangor, Malaysia, for *P*=0.6610 where the black line, red line, and blue line represent *P* ^*∗*^=0, *P* ^*∗*^=0.3450, and *P* ^*∗*^=0.4897, respectively, over a period of 2 years.

**Figure 4:**
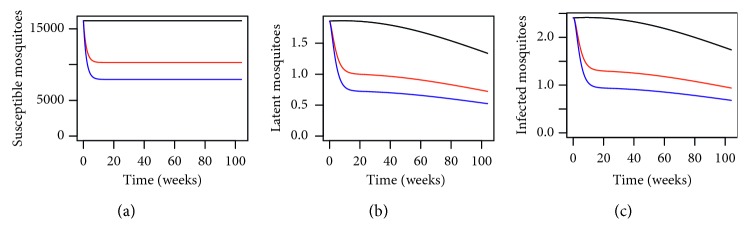
Dynamical behaviour for *Aedes* mosquitoes within the trial site in Section 15 of Shah Alam, Selangor, Malaysia, for *P*=0.6610 where the black line, red line, and blue line represent *P* ^*∗*^=0, *P* ^*∗*^=0.3450, and *P* ^*∗*^=0.4897, respectively, over a period of 2 years.

In this example, let us choose a higher *P* value, namely, 0.6610. By carrying out the same procedure as we did in Example 1, the results are given in Figures 3 and 4. From Figure 3, we can see that by using the MHSs in the trial site in Shah Alam, the number of infected individuals, the number of incidence cases, and the total dengue cases have reduced. However, if we compare the percentage of reduction to Figure 1 when we have *P*=0.1280, the percentage reductions in this case in all three categories are not as drastic when we have *P*=0.6610. This finding is reasonable as a higher *P* value means that an average person spends more time outside the trial site thus getting bitten more by *Aedes* mosquitoes. As a result, it will be more likely for a person to bring in new infection to Shah Alam and thus make it harder for the MHSs to control the number of dengue cases. However, it is still important to note that when using the MHSs in the trial site even when an average person stays outside the trial site for a longer period of time, the number of dengue cases are still reduced compared to having no MHSs at all.

To further illustrate this idea, let us look at Figure 4, where again we can see clearly that the number of susceptible, infected, and latent *Aedes* mosquitoes have reduced when using the MHSs in the trial site resulting in having less *Aedes* mosquitoes available to transmit the disease. However, again, due to a higher *P* value, the relative reduction is not as great as when we have *P*=0.1280.

In this section, we have focused on analysing the effect of using different levels of MHSs in reducing the number of dengue cases in the trial site in Section 15 of Shah Alam. The results produced have been promising by showing a great reduction in the number of dengue cases as well as the number of *Aedes* mosquitoes available to transmit the disease.

### 3.2. Effect of Different *P* and *P* ^*∗*^ Values on the Number of Dengue Cases in Shah Alam

In the previous section, we noticed that by having different *P* values, in other words, different proportions of time spent outside the trial site, this will have an impact on the percentage reduction in the number of dengue cases. Therefore, in this section, we will vary both *P* and *P* ^*∗*^ values and examine closely their impact on the number of dengue cases in the trial site.

The results from deploying the MHSs in the 18 blocks of shop houses in Section 15 of Shah Alam, Selangor, Malaysia, for various *P* values and *P* ^*∗*^ values are given in Tables 4 [Table tab5] [Table tab6]–7 where their corresponding endemic equilibrium values are given in Tables 8 [Table tab9] [Table tab10]–11, together with the total number of dengue cases over the year.

From our tables of results, regardless of what our *P* value is, in other words, regardless of how much time an average person spends outside the trial site, by using the MHSs in the trial site in Shah Alam, the total number of cases, infected individuals, and incidence cases have all reduced as a result of the MHSs reducing the total *Aedes* population.

However, it is important to note that the *P* value does have an impact on the level of reduction in the number of dengue cases. In order to illustrate this scenario better, we have extracted the incidence cases per week at endemic equilibrium level for each *P* value. The results are given in Table 12. From Table 12, we can see that the MHSs have effectively reduced the number of incidence cases per week for all values of *P*, but the relative reduction decreases as *P* increases. From Table 13, we can see clearly that when we have a small *P* value, say 0.1280, in other words, an average person stays the majority of the time inside the trial site, and there is a 61.94% reduction in the number of incidence cases in the trial site when we used 340 MHSs. The same percentage is however reduced to only 12.58% when the relative time spent outside the trial site increases to *P*=0.6610.

In this section, we have been focusing on analysing the effect of using the MHSs in reducing the number of dengue cases in the trial site in Shah Alam. From the numerical simulations and the tables of results given, it is clear that by using the MHSs, the number of dengue cases has reduced. However, it is important to point out that, from our numerical analysis carried out on equations (12) and (13), the impact from the MHSs in the trial site has very little effect in reducing the number of dengue cases in Malaysia as a whole. This is not surprising as the area of the trial site in comparison with the whole of Malaysia is almost insignificant. Therefore, even though the dengue cases have been reduced drastically in the trial site, the effect is insignificant in the whole of Malaysia. This implies that in order for the MHSs to have an impact on the number of dengue cases in Malaysia and eventually lead to dengue extinction, a fraction of residences across Malaysia would need to deploy MHSs. The most important questions to ask are “What is the fraction of coverage needed in order to achieve extinction of dengue in Malaysia?” and “What will happen to the number of dengue cases if we increase the number of MHSs?” Thus in the next section, we will focus on answering these crucial questions and analysing the effect of the MHSs in controlling the spread of dengue in Malaysia as a whole. A new dengue model will also be given with a new basic reproduction number, *R*_0,Mala_^MHS^.

## 4. New Model: MHSs in Malaysia and Dengue Extinction Condition

In this section, we will include the effect of the MHSs on the differential equations for Malaysia by assuming that a fraction *x* of the residences in Malaysia are using MHSs. Thus, equations (12) becomes

(24) $$\begin{array}{c} \frac{dS_{{\text{H}}_{1}}\left(t\right)}{dt}=-abI_{{\text{v}}_{1}}\left(t\right)\frac{S_{{\text{H}}_{1}}\left(t\right)}{N_{{\text{H}}_{1}}}-\mu_{\text{H}}S_{{\text{H}}_{1}}\left(t\right)+\mu_{\text{H}}N_{{\text{H}}_{1}},\\ \frac{dI_{{\text{H}}_{1}}\left(t\right)}{dt}=abI_{{\text{v}}_{1}}\left(t\right)\frac{S_{{\text{H}}_{1}}\left(t\right)}{N_{{\text{H}}_{1}}}-\left(\mu_{\text{H}}+\gamma \right)I_{{\text{H}}_{1}}\left(t\right),\\ \frac{dR_{{\text{H}}_{1}}\left(t\right)}{dt}=\gamma I_{{\text{H}}_{1}}\left(t\right)-\mu_{\text{H}}R_{{\text{H}}_{1}}\left(t\right),\\ \frac{dS_{{\text{v}}_{1}}\left(t\right)}{dt}=-acS_{{\text{v}}_{1}}\left(t\right)\frac{I_{{\text{H}}_{1}}\left(t\right)}{N_{{\text{H}}_{1}}}-\mu_{\text{v}}S_{{\text{v}}_{1}}\left(t\right)+\mu_{\text{v}}N_{{\text{v}}_{1}}\left(1-P^{*}x\right),\\ \frac{dL_{{\text{v}}_{1}}\left(t\right)}{dt}=acS_{{\text{v}}_{1}}\left(t\right)\frac{I_{{\text{H}}_{1}}\left(t\right)}{N_{{\text{H}}_{1}}}-\mu_{\text{v}}L_{{\text{v}}_{1}}\left(t\right)-acS_{{\text{v}}_{1}}\left(t-\tau \right)\frac{I_{{\text{H}}_{1}}\left(t-\tau \right)}{N_{{\text{H}}_{1}}}e^{-\mu_{\text{v}}\tau },\\ \frac{dI_{{\text{v}}_{1}}\left(t\right)}{dt}=acS_{{\text{v}}_{1}}\left(t-\tau \right)\frac{I_{{\text{H}}_{1}}\left(t-\tau \right)}{N_{{\text{H}}_{1}}}e^{-\mu_{\text{v}}\tau }-\mu_{\text{v}}I_{{\text{v}}_{1}}\left(t\right), \end{array}$$

where *x* (0 ≤ *x* ≤ 1) represents the fraction of coverage of the MHSs in Malaysia and *P* ^*∗*^ represents the total reduction in the proportion of *Aedes* population as a result of using the MHSs in the trial site in Shah Alam. All the other parameter values are defined as before.

Throughout this section, it will also be useful to work out the dengue incidence cases in order to compare the results of using the MHSs and not using the MHSs.

Let us define the following parameters:

- *λ*_human_^Mala^ denotes the per capita force of infection for humans in Malaysia
- *λ*_Aedes_^Mala^ denotes the per capita force of infection for *Aedes* mosquitoes in Malaysia

Then, it is easy to see that

(25) $$\begin{array}{c} \lambda_{\text{human}}^{KL}=abm\frac{I_{{\text{v}}_{1}}^{*}}{N_{{\text{v}}_{1}}},\\ \lambda_{\text{Aedes}}^{KL}=ac\frac{I_{H_{1}}^{*}}{N_{H_{1}}}, \end{array}$$

where *I*_v_1__^*∗*^ and *I*_H_1__^*∗*^, respectively, are the equilibrium number of infected mosquitoes and infected humans in Malaysia.

Before we begin with our numerical analysis on the new dengue model in Malaysia with MHSs, it is also crucial for us to construct a basic reproduction number for our new model. Let us consider a human population consisting of *N*_H_ persons in Malaysia and a population of mosquitoes consisting of *N*_v_(1 − *P* ^*∗*^ *x*) females, where *P* ^*∗*^ is the effect of using the MHS and *x* (0 ≤ *x* ≤ 1) is the fraction of homes in Malaysia employing the MHSs. Then by following the same idea as in [14], we have that the reproduction number for the new model is

(26) $$\begin{array}{c} R_{0,\text{Mala}}^{\text{MHS}}=\frac{m^{*}a^{2}bce^{-\mu_{\text{v}}\tau }}{\mu_{\text{v}}\left(\mu_{H}+\gamma \right)}, \end{array}$$

where *m* ^*∗*^=(*N*_v_(1 − *P* ^*∗*^ *x*)/*N*_H_) and all the other parameter values are defined as before. Clearly, in order to get extinction of dengue in Malaysia, *R*_0,Mala_^MHS^ needs to be less than one. Thus, the condition on extinction in relation to the fraction of coverage of the MHSs in Malaysia can be expressed as follows:

(27) $$\begin{array}{c} P^{*}x>1-\frac{\mu_{\text{v}}\left(\mu_{\text{H}}+\gamma \right)}{ma^{2}bce^{-\mu_{\text{v}}\tau }},\\ x>\left(\frac{1}{P^{*}}\right)\times \left(1-\frac{\mu_{\text{v}}\left(\mu_{\text{H}}+\gamma \right)}{ma^{2}bce^{-\mu_{\text{v}}\tau }}\right). \end{array}$$

In order to demonstrate the effect of a fraction of residences in Malaysia using MHSs as well as the relationship between *x* and *P* ^*∗*^, we will look at two examples with different *P* ^*∗*^ values. Recall that *P* ^*∗*^ represents the proportion of reduction in the total *Aedes* population as a result of using the MHSs.

### 4.1. Effect of MHSs on Dengue Cases in Malaysia When *P* ^*∗*^=0.3450

Let us recall the initial conditions that we used in Section 3.1:

(28) $$\begin{array}{c} S_{{\text{H}}_{1}}\left(0\right)=27,594.529,I_{{\text{H}}_{1}}\left(0\right)=7,966.154,R_{{\text{H}}_{1}}\left(0\right)=4,397.505,\\ S_{{\text{v}}_{1}}\left(0\right)=59,741.088,L_{{\text{v}}_{1}}\left(0\right)=6,797.226,I_{{\text{v}}_{1}}\left(0\right)=8,818.462,\\ S_{{\text{H}}_{2}}\left(0\right)=7,450.523,I_{{\text{H}}_{2}}\left(0\right)=2.151,R_{{\text{H}}_{2}}\left(0\right)=1,187.326,\\ S_{{\text{v}}_{2}}\left(0\right)=16,130.09,L_{{\text{v}}_{2}}\left(0\right)=1.835,I_{{\text{v}}_{2}}\left(0\right)=2.381, \end{array}$$

where all the parameter values are defined as before.

**Figure 5:**
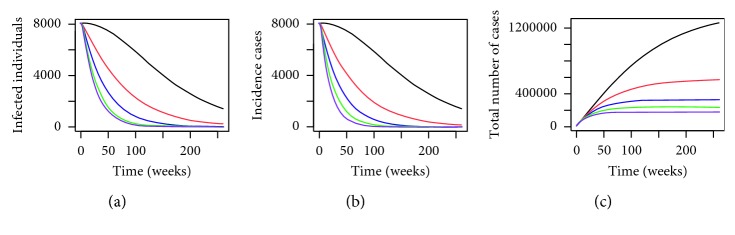
Numerical simulations for (a) infected individuals, (b) incidence cases (in weeks), and (c) total number of dengue cases in Malaysia where the black line: *x*=0, red line: *x*=0.1, blue line: *x*=0.20, green line: *x*=0.30, and purple line: *x*=0.40 over a period of 5 years where *P* ^*∗*^=0.3450.

**Figure 6:**
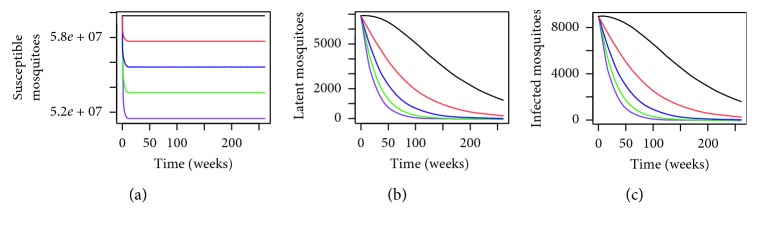
Dynamical behaviour for *Aedes* mosquitoes in Malaysia where the black line: *x*=0, red line: *x*=0.1, blue line: *x*=0.20, green line: *x*=0.30, and purple line: *x*=0.40 over a period of 5 years where *P* ^*∗*^=0.3450.

From Figure 5, we can see that a fraction of residences in Malaysia deploying MHSs has effectively reduced the number of infected individuals, dengue incidence cases, and the total number of dengue cases. The higher the *x* value, in other words, the greater the fraction of homes that we have in Malaysia that use the MHSs, the greater the reduction in the number of dengue cases, which is to be expected. This finding is again confirmed in Figure 6, where we can see that the number of susceptible, infected, and latent *Aedes* mosquitoes in Malaysia have reduced as a result of deploying a fraction of MHSs in Malaysia.

In addition, if we look at the blue, green, and purple lines in Figure 5, the number of infected individuals and incidence cases tends to zero. Thus from the simulations, it appears that dengue cases have been eliminated when we have around 20% to 40% coverage of MHSs in Malaysia. This is a very promising result as the simulations not only indicate the effectiveness of MHSs in reducing the number of dengue cases in Malaysia but also show that by having the right level of coverage, dengue can be eradicated. In order to identify the exact percentage coverage needed to achieve extinction of dengue in Malaysia when *P* ^*∗*^=0.3450, let us refer to the extinction criterion given by using equation (27) mentioned at the start of this section. Recall that

(29) $$\begin{array}{c} x>\left(\frac{1}{P^{*}}\right)\times \left(1-\frac{\mu_{\text{v}}\left(\mu_{\text{H}}+\gamma \right)}{ma^{2}bce^{-\mu_{\text{v}}\tau }}\right). \end{array}$$

By substituting all the required parameter values into the right-hand side of equation (29), we have that in order to eliminate dengue in Malaysia when *P* ^*∗*^=0.3450, the fraction of coverage of MHSs needs to be greater than 0.4043. In other words, around 40.43% of the homes in Malaysia would need to use this level of MHSs in order to eradicate dengue in Malaysia.

From Table 14, we have the corresponding number of dengue cases per week at the endemic equilibrium level in Malaysia with different *x* values and *P* ^*∗*^=0.3450. From the table, the results clearly support our extinction threshold of needing *x* > 0.4043 for dengue extinction in Malaysia. As the *x* value increases, the number of dengue incidence cases also decreases.

In this section, we focused on analysing the effect of having *P* ^*∗*^=0.3450 and we found that to achieve extinction in Malaysia, we need *x* > 0.4043. In the next section, we will observe what happens when we increase *P* ^*∗*^ to 0.4897.

### 4.2. Effect of MHSs on Dengue Cases in Malaysia When *P* ^*∗*^=0.4897

**Figure 7:**
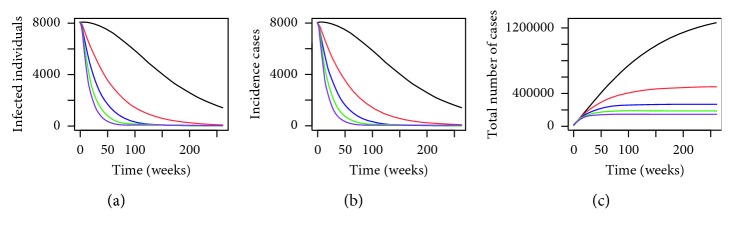
Numerical simulations for (a) infected individuals, (b) incidence cases (in weeks), and (c) total number of dengue cases in Malaysia where the black line: *x*=0, red line: *x*=0.1, blue line: *x*=0.20, green line: *x*=0.30, and purple line: *x*=0.40 over a period of 5 years where *P* ^*∗*^=0.4897.

**Figure 8:**
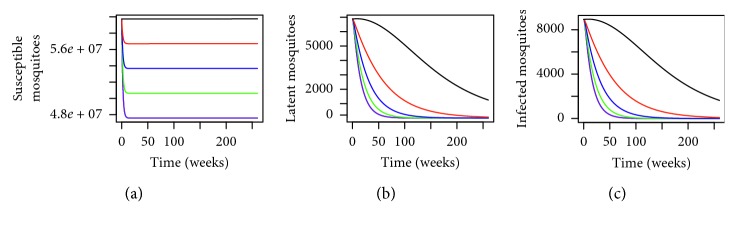
Dynamical behaviour for *Aedes* mosquitoes in Malaysia where the black line: *x*=0, red line: *x*=0.1, blue line: *x*=0.20, green line: *x*=0.30, and purple line: *x*=0.40 over a period of 5 years where *P* ^*∗*^=0.4897.

The numerical simulations for the number of infected individuals, number of incidence cases, and the total number of dengue cases in Malaysia when we have *P* ^*∗*^=0.4897 for different levels of coverage are given in Figure 7. From the simulation, it is clear that using the MHSs throughout Malaysia has effectively reduced the number of dengue cases. From Figure 8, we can also see the reduction in the number of susceptible, latent, and infected *Aedes* mosquitoes as a result of the MHSs.

By comparing Figure 5 with Figure 7, we can see that for a higher value of *P* ^*∗*^, the curves for infected individuals, incidence cases, and total number of cases reduce more steeply. This is again to be expected as by having a higher *P* ^*∗*^ value, we would expect the effect of reducing dengue cases to be more significant.

Similarly to Section 4.1, by substituting the appropriate parameter values in the right-hand side of the extinction criteria given in equation (4), we have that for *P* ^*∗*^=0.4897, in order to achieve extinction in dengue in Malaysia, *x* needs to be greater than 0.2848. In other words, when *P* ^*∗*^=0.4897, we would need to have around 28.48% coverage of MHSs in Malaysia. Note that the percentage of coverage in this case is lower than the one needed in Section 4.1 for extinction.

From Table 15, we can clearly see that the results support our extinction criteria, by showing the number of dengue incidence cases in both Malaysia and in the trial site in Section 15 of Shah Alam to be zero when the fraction of coverage in Malaysia is greater than 28.48%.

### 4.3. Extinction Criteria in terms of *x* and *P* ^*∗*^

In the previous two sections, we focused on answering the question “What proportion of homes in Malaysia need to use MHSs if the proportion of reduction in the *Aedes* population is a given value of *P* ^*∗*^ (say 0.3450 or 0.4897)?” In this section, we shall instead aim to answer the question “What is the proportion of reduction needed in the *Aedes* population from the MHSs if we wish to achieve dengue extinction in Malaysia by a fraction *x* of homes using MHSs?”

By doing simple algebraic rearrangement in the extinction condition given in equation (27), we have the required answers given in Table 16. We can interpret the results from Table 16 as if we wish to achieve extinction in dengue in Malaysia by only covering 15% of Malaysia with MHSs; then the effect from using the MHSs needs to be able to reduce the total *Aedes* population by about 93.1%. However, if we can cover 40% of Malaysia with MHSs, then extinction is possible as long as the MHSs are able to reduce the *Aedes* population by 34.9%. This information will be useful in helping us in deciding the right amount of MHSs that we need to deploy in Malaysia in order to reduce or eliminate dengue.

## 5. Discussion and Conclusion

Dengue is endemic in Malaysia and has caused the Malaysian government much money every year in trying to control its spread. In this paper, we have used mathematical modelling to analyse the spread of dengue between humans and *Aedes* mosquitoes and evaluated the effectiveness of using a type of autodissemination trap, the Mosquito Home System (MHS), in reducing the number of dengue cases. By using the MHS trial data obtained from the trial site in Section 15 of Shah Alam, Selangor, Malaysia, we are able to produce numerical simulations and results which highlighted the effectiveness of using the MHSs in reducing the number of dengue cases in the trial site. From the numerical results produced in *R*, we can see that the MHSs drastically reduced the number of susceptible, infected, and latent *Aedes* mosquitoes at the trial site.

Later on, we found that from the numerical simulations that having MHSs in the trial site in Shah Alam alone has little effect in controlling the dengue cases occurring in the whole of Malaysia, which is not surprising. Therefore, we improved on our model by including the effect of MHSs in the differential equations for Malaysia by assuming that a fraction *x* of the homes in Malaysia employ MHSs. From our numerical simulations produced for the dengue model for Malaysia, we have found that the MHSs have effectively reduced the number of dengue cases. A basic reproduction number, *R*_0,Mala_^MHS^, for this new model was obtained which allowed us to construct the crucial extinction condition which we need to meet in order to eliminate dengue in Malaysia. Finally, we obtained the required fraction of coverage of MHSs that is needed in order to obtain extinction. These results should help us in deciding on the right level of MHSs coverage that is needed in Malaysia in order to better control the spread of dengue.

The analytical work that has been performed in this paper is based on the data from the site of the MHS trial taking place in Section 15 of Shah Alam. It is reasonable to think that depending on the location and district of the trial site, the MHSs would perform differently. Therefore, if we are able to have more trial data in the future, we could extend our dengue model by taking into consideration the effect of MHSs from different districts in Malaysia.

## Figures and Tables

**Table 1:** Parameter values given in equation (1).

Parameter values	Biological meanings
*a*	*Aedes* mosquitoes biting rate
*b*	Probability of transmission of dengue when an infectious mosquito bites a susceptible human
*c*	Probability of transmission of dengue when a susceptible mosquito bites an infectious human
*N*_H_	Human population
*μ*_H_	Per capita human mortality rate
*γ*	Per capita human recovery rate
*μ*_v_	Per capita mortality rate for *Aedes* mosquitoes
*N*_v_	*Aedes* mosquitoe population
*τ*	Dengue extrinsic incubation period

**Table 2:** Proportions of *Aedes* bites according to biting activities at different time intervals.

Time outside the trial site	*P*	1 − *P*
6 a.m. to 7.45 p.m.	0.6610	0.3390
6 a.m. to 6.45 p.m.	0.6210	0.3790
9 a.m. to 7.45 p.m.	0.4770	0.5230
8 a.m. to 5.45 p.m.	0.3190	0.6810
9 a.m. to 4.45 p.m.	0.1280	0.8720

**Table 3:** Parameter values (*N*_H_1__ based on 2017) for equations (12) and (13).

Parameter	Values
*a*	0.20/day
*b*	0.75 [26]
*c*	0.375 [26]
*N*_H_1__	32,000,000 [22]
*N*_H_2__	8,640
*μ*_H_	0.0000366/day [27]
*γ*	1/7/day [11]
*μ*_v_	1/14/day [28]
*N*_v_1__	*m* × *N*_H_1__
*N*_v_2__	*m* × *N*_H_2__
*τ*	8 days [11]

**Table 4:** Mean values for variables within the trial site in Section 15 of Shah Alam, Selangor, Malaysia, for *P*=0.1280, where *P* ^*∗*^=0: no MHSs, *P* ^*∗*^=0.3450: 340 MHSs, and *P* ^*∗*^=0.4897: 625 MHSs to 3 d.p after 1 year. Note that the total number of cases refers to the cumulative incidence of the number of dengue cases over the year.

*P*=0.1280								
*P* ^*∗*^ values	*S*_H_2__(*t*)	*I*_H_2__(*t*)	*R*_H_2__(*t*)	Incidence cases	Total number of cases	*S*_v_2__(*t*)	*L*_v_2__(*t*)	*I*_v_2__(*t*)
0	7,403.097	2.118	1,234.785	2.116	127.862	16,130.150	1.808	2.352
0.3450	7,428.509	0.973	1,210.518	0.944	66.900	10,780.590	0.582	0.853
0.4897	7,433.486	0.791	1,205.723	0.759	57.280	8,536.153	0.393	0.617

**Table 5 tab5:** Mean values for variables within the trial site in Section 15 of Shah Alam, Selangor, Malaysia, for *P*=0.3190, where *P* ^*∗*^=0: no MHSs, *P* ^*∗*^=0.3450: 340 MHSs, and *P* ^*∗*^=0.4897: 625 MHSs to 3 d.p. after 1 year. Note that the total number of cases refers to the cumulative incidence of the number of cases over the year.

*P*=0.3190								
*P* ^*∗*^ values	*S*_H_2__(*t*)	*I*_H_2__(*t*)	*R*_H_2__(*t*)	Incidence cases	Total number of cases	*S*_v_2__(*t*)	*L*_v_2__(*t*)	*I*_v_2__(*t*)
0	7,403.097	2.118	1,234.785	2.116	127.862	16,130.150	1.808	2.352
0.3450	7,419.777	1.390	1,218.834	1.371	89.134	10,780.080	0.812	1.131
0.4897	7,424.149	1.215	1,214.636	1.193	79.871	8,535.748	0.576	0.840

**Table 6 tab6:** Mean values for variables within the trial site in Section 15 of Shah Alam, Selangor, Malaysia, for *P*=0.4770, where *P* ^*∗*^=0: no MHSs, *P* ^*∗*^=0.3450: 340 MHSs, and *P* ^*∗*^=0.4897: 625 MHSs to 3 d.p after 1 year. Note that the total number of cases refers to the cumulative incidence of the number of cases over the year.

*P*=0.4770								
*P* ^*∗*^ values	*S*_H_2__(*t*)	*I*_H_2__(*t*)	*R*_H_2__(*t*)	Incidence cases	Total number of cases	*S*_v_2__(*t*)	*L*_v_2__(*t*)	*I*_v_2__(*t*)
0	7,403.097	2.118	1,234.785	2.116	127.862	16,130.150	1.808	2.352
0.3450	7,414.394	1.634	1,223.973	1.621	102.135	10,779.780	0.947	1.295
0.4897	7,417.874	1.491	1,220.635	1.476	94.581	8,535.483	0.695	0.985

**Table 7:** Mean values for variables within the trial site in Section 15 of Shah Alam, Selangor, Malaysia, for *P*=0.6610, where *P* ^*∗*^=0: no MHSs, *P* ^*∗*^=0.3450: 340 MHSs, and *P* ^*∗*^=0.4897: 625 MHSs to 3 d.p after 1 year. Note that the total number of cases refers to the cumulative incidence of the number of cases over the year.

*P*=0.6610								
*P* ^*∗*^ values	*S*_H_2__(*t*)	*I*_H_2__(*t*)	*R*_H_2__(*t*)	Incidence cases	Total number of cases	*S*_v_2__(*t*)	*L*_v_2__(*t*)	*I*_v_2__(*t*)
0	7,403.097	2.118	1,234.785	2.116	127.862	16,130.150	1.808	2.352
0.3450	7,409.527	1.847	1,228.627	1.839	113.464	10,779.52	1.065	1.438
0.4897	7,411.799	1.753	1,226.448	1.744	108.484	8,535.232	0.808	1.123

**Table 8:** Endemic equilibrium values for variables within the trial site in Section 15 of Shah Alam, Selangor, Malaysia, for *P*=0.1280, where *P* ^*∗*^=0: no MHSs, *P* ^*∗*^=0.3450: 340 MHSs, and *P* ^*∗*^=0.4897: 625 MHSs to 3 d.p.

*P*=0.1280								
*P* ^*∗*^ values	*S*_H_2__(*t*)	*I*_H_2__(*t*)	*R*_H_2__(*t*)	Incidence cases	Total number of cases	*S*_v_2__(*t*)	*L*_v_2__(*t*)	*I*_v_2__(*t*)
0	7,433.524	0.310	1,206.165	0.310	16.129	16,133.700	0.265	0.343
0.3450	8,182.197	0.118	457.685	0.118	6.120	10,567.820	0.066	0.085
0.4897	8,297.496	0.088	342.416	0.088	4.579	8,233.250	0.038	0.050

**Table 9 tab9:** Endemic equilibrium values for variables within the trial site in Section 15 of Shah Alam, Selangor, Malaysia, for *P*=0.3190, where *P* ^*∗*^=0: no MHSs, *P* ^*∗*^=0.3450: 340 MHSs, and *P* ^*∗*^=0.4897: 625 MHSs to 3 d.p.

*P*=0.3190								
*P* ^*∗*^ values	*S*_H_2__(*t*)	*I*_H_2__(*t*)	*R*_H_2__(*t*)	Incidence cases	Total number of cases	*S*_v_2__(*t*)	*L*_v_2__(*t*)	*I*_v_2__(*t*)
0	7,433.524	0.310	1,206.165	0.310	16.129	16,133.700	0.265	0.343
0.3450	7,868.245	0.198	771.557	0.198	10.318	10,567.720	0.111	0.144
0.4897	7,980.868	0.169	658.963	0.169	8.812	8,233.169	0.074	0.096

**Table 10 tab10:** Endemic equilibrium values for variables within the trial site in Section 15 of Shah Alam, Selangor, Malaysia, for *P*=0.4770, where *P* ^*∗*^=0: no MHSs, *P* ^*∗*^=0.3450: 340 MHSs, and *P* ^*∗*^=0.4897: 625 MHSs to 3 d.p.

*P*=0.4770								
*P* ^*∗*^ values	*S*_H_2__(*t*)	*I*_H_2__(*t*)	*R*_H_2__(*t*)	Incidence cases	Total number of cases	*S*_v_2__(*t*)	*L*_v_2__(*t*)	*I*_v_2__(*t*)
0	7,433.524	0.310	1,206.165	0.310	16.129	16,133.700	0.265	0.343
0.3450	7,713.490	0.238	926.272	0.238	12.387	10,567.670	0.133	0.173
0.4897	7,801.087	0.216	838.698	0.216	11.215	8,233.123	0.094	0.122

**Table 11:** Endemic equilibrium values for variables within the trial site in Section 15 of Shah Alam, Selangor, Malaysia, for *P*=0.6610, where *P* ^*∗*^=0: no MHSs, *P* ^*∗*^=0.3450: 340 MHSs, and *P* ^*∗*^=0.4897: 625 MHSs to 3 d.p.

*P*=0.6610								
*P* ^*∗*^ values	*S*_H_2__(*t*)	*I*_H_2__(*t*)	*R*_H_2__(*t*)	Incidence cases	Total number of cases	*S*_v_2__(*t*)	*L*_v_2__(*t*)	*I*_v_2__(*t*)
0	7,433.524	0.310	1,206.165	0.310	16.129	16,133.700	0.265	0.343
0.3450	7,587.452	0.271	1,052.277	0.271	14.072	10,567.630	0.151	0.196
0.4897	7,642.698	0.256	997.045	0.256	13.333	8,233.082	0.112	0.145

**Table 12:** Number of incidence cases per week for different numbers of MHSs within the trial site in Shah Alam, Selangor, Malaysia, with proportions of time spent outside the trial site at the EE.

*P* values	0 MHSs	340 MHSs	625 MHSs
0.1280	0.310	0.118	0.088
0.3190	0.310	0.198	0.169
0.4770	0.310	0.238	0.216
0.6610	0.310	0.271	0.256

**Table 13:** Percentage reduction in the number of incidence cases at EE for various values of *P*.

*P* values	*P* ^*∗*^=0 to *P* ^*∗*^=0.3450 (%)	*P* ^*∗*^=0.3450 to *P* ^*∗*^=0.4897 (%)
0.1280	61.94	24.48
0.3190	36.13	14.65
0.4770	23.23	9.24
0.6610	12.58	5.54

**Table 14:** Dengue incidence cases per week at EE for different fractions of coverage of MHSs in Malaysia where *P* ^*∗*^=0.3450: 340 MHSs to 3 d.p.

*P* ^*∗*^=0.3450	
*x* values	Dengue incidence cases in Malaysia
0	1,148.814
0.10	895.947
0.20	624.341
0.30	331.869
0.39	24.278
0.41	0.0

**Table 15:** Dengue incidence cases per week at EE for different fractions of coverage of MHSs in Malaysia where *P* ^*∗*^=0.4897: 625 MHSs to 3 d.p.

*P* ^*∗*^=0.4897	
*x* values	Dengue incidence cases in Malaysia
0	1,148.813
0.10	784.437
0.20	380.510
0.25	161.697
0.29	0.0

**Table 16:** Proportion of reduction needed in the *Aedes* mosquitoes population from using MHSs to achieve dengue extinction in Malaysia for different *x* values to 3 d.p.

*x* value	*P* ^*∗*^ value
0.05	Not possible
0.10	Not possible
0.15	0.931
0.20	0.698
0.25	0.559
0.30	0.466
0.35	0.399
0.40	0.349
0.45	0.303

## Data Availability

The data used to support the findings of this study are available from the corresponding author upon request.
